# Complete genome sequence of *Bacteroides salanitronis* type strain (BL78^T^)

**DOI:** 10.4056/sigs.1704212

**Published:** 2011-04-29

**Authors:** Sabine Gronow, Brittany Held, Susan Lucas, Alla Lapidus, Tijana Glavina Del Rio, Matt Nolan, Hope Tice, Shweta Deshpande, Jan-Fang Cheng, Sam Pitluck, Konstantinos Liolios, Ioanna Pagani, Natalia Ivanova, Konstantinos Mavromatis, Amrita Pati, Roxane Tapia, Cliff Han, Lynne Goodwin, Amy Chen, Krishna Palaniappan, Miriam Land, Loren Hauser, Yun-Juan Chang, Cynthia D. Jeffries, Evelyne-Marie Brambilla, Manfred Rohde, Markus Göker, John C. Detter, Tanja Woyke, James Bristow, Victor Markowitz, Philip Hugenholtz, Nikos C. Kyrpides, Hans-Peter Klenk, Jonathan A. Eisen

**Affiliations:** 1 DSMZ - German Collection of Microorganisms and Cell Cultures GmbH, Braunschweig, Germany; 2 DOE Joint Genome Institute, Walnut Creek, California, USA; 3 Los Alamos National Laboratory, Bioscience Division, Los Alamos, New Mexico, USA; 4 Biological Data Management and Technology Center, Lawrence Berkeley National Laboratory, Berkeley, California, USA; 5 Lawrence Livermore National Laboratory, Livermore, California, USA; 6 HZI – Helmholtz Centre for Infection Research, Braunschweig, Germany; 7 University of California Davis Genome Center, Davis, California, USA; 8 Australian Centre for Ecogenomics, School of Chemistry and Molecular Biosciences, The University of Queensland, Brisbane, Australia

**Keywords:** strictly anaerobic, non-motile, rod-shaped, Gram-negative, mesophilic, cecum, poultry, chemoorganotrophic, *Bacteroidaceae*, GEBA

## Abstract

*Bacteroides salanitronis* Lan *et al.* 2006 is a species of the genus *Bacteroides*, which belongs to the family *Bacteroidaceae*. The species is of interest because it was isolated from the gut of a chicken and the growing awareness that the anaerobic microflora of the cecum is of benefit for the host and may impact poultry farming. The 4,308,663 bp long genome consists of a 4.24 Mbp chromosome and three plasmids (6 kbp, 19 kbp, 40 kbp) containing 3,737 protein-coding and 101 RNA genes and is a part of the *** G****enomic* *** E****ncyclopedia of* *** B****acteria and* *** A****rchaea * project.

## Introduction

Strain BL78^T^ (= DSM 18170 = CCUG 54637 = JCM 13657) is the type strain of *Bacteroides salanitronis* which belongs to the large genus *Bacteroides* [1,2]. Currently, there are 88 species placed in the genus *Bacteroides*. The species epithet is derived from the name of Joseph P. Salanitro, an American microbiologist. *B. salanitronis* strain BL78^T^ was isolated among other *Bacteroides* strains from the cecum of a healthy chicken. No other strain belonging to the same species has been identified [2]. Many *Bacteroides* species are common inhabitants of the intestine where they help to degrade complex molecules such as polysaccharides or transform steroids [3,4]. They also play a role as beneficent protectors of the gut against pathogenic microorganisms [5]. Here we present a summary classification and a set of features for *B. salanitronis* BL78^T^, together with the description of the complete genomic sequencing and annotation.

## Classification and features

A representative genomic 16S rRNA sequence of strain BL78^T^ was compared using NCBI BLAST under default settings (e.g., considering only the high-scoring segment pairs (HSPs) from the best 250 hits) with the most recent release of the Greengenes database [6] and the relative frequencies, weighted by BLAST scores, of taxa and keywords (reduced to their stem [7]) were determined. The single most frequent genus was *Bacteroides* (100.0%) (1 hit in total). Regarding the single hit to sequences from members of the species, the average identity within HSPs was 99.7%, whereas the average coverage by HSPs was 96.2%. No hits to sequences with (other) species names were found. The highest-scoring environmental sequence was DQ456041 ('pre-adolescent turkey cecum clone CFT112F11'), which showed an identity of 96.8% and an HSP coverage of 63.9%. The five most frequent keywords within the labels of environmental samples which yielded hits were 'fecal' (9.3%), 'microbiota' (7.5%), 'human' (7.1%), 'antibiot, effect, gut, pervas' (7.1%) and 'anim, beef, cattl, coli, escherichia, feedlot, habitat, synecolog' (2.2%) (249 hits in total).

**Figure 1:**
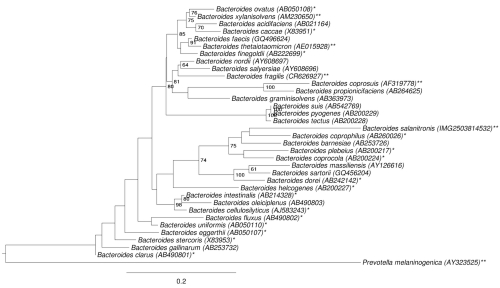
Phylogenetic tree highlighting the position of *B. salanitronis* relative to a selection of other type strains within the genus. The tree was inferred from 1,412 aligned characters [8,9] of the 16S rRNA gene sequence under the maximum likelihood criterion [10] and rooted in accordance with the current taxonomy. The branches are scaled in terms of the expected number of substitutions per site. Numbers to the right of bifurcations are support values from 1,000 bootstrap replicates [11] if larger than 60%. Lineages with type strain genome sequencing projects registered in GOLD [12] but unpublished are labeled with one asterisk, published genomes with two asterisks [13-15].

Figure 1 shows the phylogenetic neighborhood of *B. salanitronis* in a 16S rRNA based tree. The sequences of the six 16S rRNA gene copies in the genome differ from each other by up to 26 nucleotides, and differ by up to 26 nucleotides from the previously published 16S rRNA sequence (AB253731).

**Figure 2:**
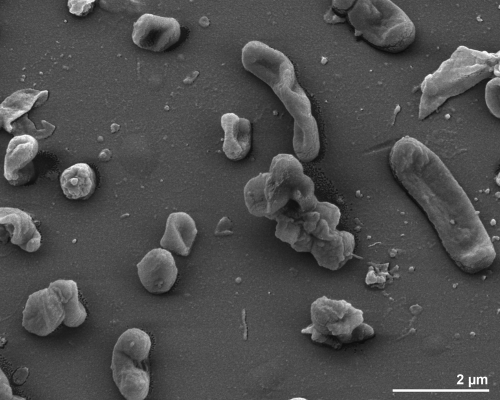
Scanning electron micrograph of *B. salanitronis* BL78^T^

The cells of *B. salanitronis* are generally rod-shaped (0.4-0.7 × 0.8-5.6 µm) with rounded ends (Figure 2). The cells are usually arranged singly or in pairs [2]. *B. salanitronis* is a Gram-negative, non-spore-forming bacterium (Table 1) that is described as non-motile, with only five genes associated with motility having been found in the genome (see below). The temperature optimum for strain BL78^T^ is 37°C. *B. salanitronis* is a strictly anaerobic chemoorganotroph and is able to ferment glucose, mannose, sucrose, maltose, arabinose, cellobiose, lactose, xylose and raffinose [2]. The organism hydrolyzes esculin but does not liquefy gelatin, and neither reduces nitrate nor produces indole from tryptophan [2]. *B. salanitronis* does not utilize trehalose, glycerol, mannitol, sorbitol or melezitose; rhamnose and salicin are fermented weakly [2]. Growth is possible in the presence of bile [2]. Major fermentation products from broth (1% peptone, 1% yeast extract, and 1% glucose each (w/v)) are acetic acid and succinic acid, whereas isovaleric acid is produced in small amounts [2]. *B. salanitronis* shows activity for alkaline phosphatase, α- and β-galactosidases, α- and β-glucosidases, α-arabinosidase, leucyl glycine arylamidase, alanine arylamidase and glutamyl glutamic acid arylamidase but no activity for urease, catalase, glutamic acid decarboxylase, arginine dihydrolase, β-galactosidase 6-phosphate, β-glucuronidase, *N*-acetyl-β-glucosaminidase, α-fucosidase and arginine, proline, leucine, phenylalanine, pyroglutamic acid, tyrosine, glycine, histidine and serine arylamidase [2].

**Table 1:** Classification and general features of *B. salanitronis* BL78^T^ according to the MIGS recommendations [16].

MIGS ID	Property	Term	Evidence code
	Current classification	Domain *Bacteria*	TAS [17]
		Phylum '*Bacteroidetes*'	TAS [18]
		Class '*Bacteroidia*'	TAS [19]
		Order '*Bacteroidales*'	TAS [20]
		Family *Bacteroidaceae*	TAS [21,22]
		Genus *Bacteroides*	TAS [21,23-26]
		Species *Bacteroides salanitronis*	TAS [2]
		Type strain BL78	TAS [2]
	Gram stain	negative	TAS [2]
	Cell shape	rod-shaped	TAS [2]
	Motility	non-motile	TAS [2]
	Sporulation	none	TAS [2]
	Temperature range	mesophile	TAS [2]
	Optimum temperature	37°C	TAS [2]
	Salinity	normal	NAS
MIGS-22	Oxygen requirement	strictly anaerobic	TAS [2]
	Carbon source	carbohydrates	TAS [2]
	Energy metabolism	chemoorganotroph	TAS [2]
MIGS-6	Habitat	chicken	TAS [2]
MIGS-15	Biotic relationship	free-living	NAS
MIGS-14	Pathogenicity	none	NAS
	Biosafety level	1	TAS [27]
	Isolation	chicken cecum	TAS [2]
MIGS-4	Geographic location	Japan	TAS [2]
MIGS-5	Sample collection time	November 2005	IDA
MIGS-4.1	Latitude	not reported	
MIGS-4.2	Longitude	not reported	
MIGS-4.3	Depth	not reported	
MIGS-4.4	Altitude	not reported	

Evidence codes - IDA: Inferred from Direct Assay (first time in publication); TAS: Traceable Author Statement (i.e., a direct report exists in the literature); NAS: Non-traceable Author Statement (i.e., not directly observed for the living, isolated sample, but based on a generally accepted property for the species, or anecdotal evidence). These evidence codes are from of the Gene Ontology project [28]. If the evidence code is IDA, then the property was directly observed by one of the authors or an expert mentioned in the acknowledgements.

### Chemotaxonomy

*B. salanitronis* strain BL78^T^ contains menaquinones MK-11 and MK-12 as principal respiratory quinones (43% each), small amounts of MK-10 (5%) and MK-13 (7%) are found as minor components [2]. The major fatty acids found were *anteiso-*C_15:0_ (32%), *iso-*C_15:0_ (14%), 3-hydroxy C_16:0_ (12%) and 3-hydroxy *iso*-C_17:0_ (10%). Fatty acids C_14:0_ (4%), C_15:0_ (2%), C_16:0_ (8%), C_18:1_ (2%), C_18:2_ (2%) and *iso*-C_14:0_ (2%) were found in minor amounts [2].

## Genome sequencing and annotation

### Genome project history

This organism was selected for sequencing on the basis of its phylogenetic position [29], and is part of the *** G****enomic* *** E****ncyclopedia of* *** B****acteria and* *** A****rchaea * project [30]. The genome project is deposited in the Genomes On Line Database [31] and the complete genome sequence is deposited in GenBank. Sequencing, finishing and annotation were performed by the DOE Joint Genome Institute (JGI). A summary of the project information is shown in Table 2.

**Table 2:** Genome sequencing project information

**MIGS ID**	**Property**	**Term**
MIGS-31	Finishing quality	Finished
MIGS-28	Libraries used	Three genomic libraries: one 454 pyrosequence standard library, one 454 PE library (7 kb insert size), one Illumina library
MIGS-29	Sequencing platforms	Illumina GAii, 454 GS FLX Titanium
MIGS-31.2	Sequencing coverage	283.0 × Illumina; 37.7 × pyrosequence
MIGS-30	Assemblers	Newbler version 2.3-PreRelease-09-14-2009-bin, Velvet, phrap version SPS 4.24
MIGS-32	Gene calling method	Prodigal 1.4, GenePRIMP
	INSDC ID	CP002530 (chromosome) CP002531 (plasmid 1) CP002532 (plasmid 2) CP002533 (plasmid 3)
	Genbank Date of Release	February 28, 2011
	GOLD ID	Gc001665
	NCBI project ID	40066
	Database: IMG-GEBA	2503754023
MIGS-13	Source material identifier	DSM 18170
	Project relevance	Tree of Life, GEBA

### Growth conditions and DNA isolation

*B. salanitronis* BL78^T^, DSM 18170, was grown anaerobically in DSMZ medium 104 (Peptone-Yeast extract-Glucose broth) [32] at 37°C. DNA was isolated from 0.5-1 g of cell paste using MasterPure Gram-positive DNA purification kit (Epicentre MGP04100) following the standard protocol as recommended by the manufacturer, adding 20 µL lysozyme (100mg/µl), and 10 µL mutanolysin, achromopeptidase, and lysostaphine, each, for 40 min lysis at 37ºC followed by one hour incubation on ice. DNA is available through the DNA Bank Network [33].

### Genome sequencing and assembly

The genome was sequenced using a combination of Illumina and 454 sequencing platforms. All general aspects of library construction and sequencing can be found at the JGI website [34]. Pyrosequencing reads were assembled using the Newbler assembler version 2.3-PreRelease-09-14-2009-bin (Roche). The initial Newbler assembly consisting of 100 contigs in two scaffolds was converted into a phrap assembly [35] by making fake reads from the consensus, to collect the read pairs in the 454 paired-end library. Illumina GAii sequencing data (920.8 Mb) was assembled with Velvet, version 0.7.63 [36] and the consensus sequences were shredded into 1.5 kb overlapped fake reads and assembled together with the 454 data. The 454 draft assembly was based on 109.0 Mb of 454 standard data and all of the 454 paired end data. Newbler parameters are -consed -a 50 -l 350 -g -m -ml 20. The Phred/Phrap/Consed software package [35] was used for sequence assembly and quality assessment in the subsequent finishing process. After the shotgun stage, reads were assembled with parallel phrap (High Performance Software, LLC). Possible mis-assemblies were corrected with gapResolution [34], Dupfinisher [37], or sequencing cloned bridging PCR fragments with subcloning or transposon bombing (Epicentre Biotechnologies, Madison, WI). Gaps between contigs were closed by editing in Consed, by PCR and by Bubble PCR primer walks (J.-F.Chang, unpublished). A total of 193 additional reactions and four shatter libraries were necessary to close gaps and to raise the quality of the finished sequence. Illumina reads were also used to correct potential base errors and increase consensus quality using a software Polisher developed at JGI [38]. The error rate of the completed genome sequence is less than 1 in 100,000. Together, the combination of the Illumina and 454 sequencing platforms provided 320.7 × coverage of the genome. The final assembly contained 393,135 pyrosequence and 25,576,764 Illumina reads.

### Genome annotation

Genes were identified using Prodigal [39] as part of the Oak Ridge National Laboratory genome annotation pipeline, followed by a round of manual curation using the JGI GenePRIMP pipeline [40]. The predicted CDSs were translated and used to search the National Center for Biotechnology Information (NCBI) nonredundant database, UniProt, TIGR-Fam, Pfam, PRIAM, KEGG, COG, and InterPro databases. Additional gene prediction analysis and functional annotation was performed within the Integrated Microbial Genomes - Expert Review (IMG-ER) platform [41].

## Genome properties

**Figure 3:**
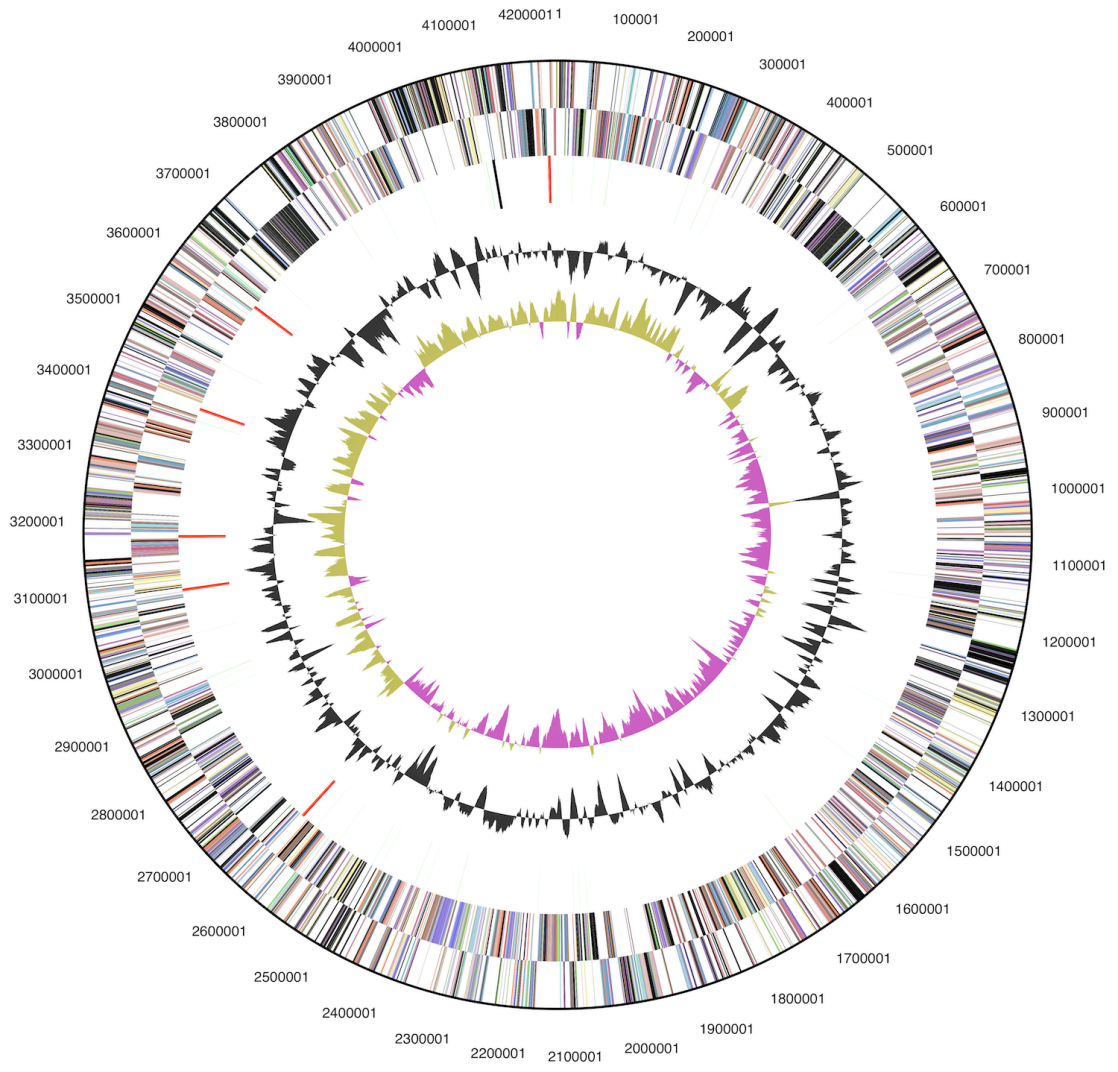
Graphical circular map of the chromosome (plasmid maps not shown). From outside to the center: Genes on forward strand (color by COG categories), Genes on reverse strand (color by COG categories), RNA genes (tRNAs green, rRNAs red, other RNAs black), GC content, GC skew.

The genome consists of a 4,242,803 bp long chromosome with a G+C content of 47%, as well as three plasmids of 6,277 bp, 18,280 bp and 40,303 bp length (Table 3 and Figure 3). Of the 3,838 genes predicted, 3,737 were protein-coding genes, and 101 RNAs; 96 pseudogenes were also identified. The majority of the protein-coding genes (57.3%) were assigned with a putative function while the remaining ones were annotated as hypothetical proteins. The distribution of genes into COGs functional categories is presented in Table 4.

**Table 3:** Genome Statistics

**Attribute**	**Value**	**% of Total**
Genome size (bp)	4,308,663	100.00%
DNA coding region (bp)	3,759,354	87.25%
DNA G+C content (bp)	2,003,128	46.49%
Number of replicons	4	
Extrachromosomal elements	3	
Total genes	3,838	100.00%
RNA genes	101	2.63%
rRNA operons	6	
Protein-coding genes	3,737	97.37%
Pseudo genes	96	2.50%
Genes with function prediction	2,200	57.32%
Genes in paralog clusters	876	22.82%
Genes assigned to COGs	2,013	52.45%
Genes assigned Pfam domains	2,269	59.12%
Genes with signal peptides	918	23.92%
Genes with transmembrane helices	794	20.69%
CRISPR repeats	0	

**Table 4:** Number of genes associated with the general COG functional categories

Code	value	%age	Description
J	147	6.8	Translation, ribosomal structure and biogenesis
A	0	0.0	RNA processing and modification
K	143	6.6	Transcription
L	194	9.0	Replication, recombination and repair
B	0	0.0	Chromatin structure and dynamics
D	31	1.4	Cell cycle control, cell division, chromosome partitioning
Y	0	0.0	Nuclear structure
V	63	2.9	Defense mechanisms
T	85	3.9	Signal transduction mechanisms
M	193	8.9	Cell wall/membrane/envelope biogenesis
N	5	0.2	Cell motility
Z	0	0.0	Cytoskeleton
W	0	0.0	Extracellular structures
U	61	2.8	Intracellular trafficking, secretion, and vesicular transport
O	61	2.8	Posttranslational modification, protein turnover, chaperones
C	105	4.9	Energy production and conversion
G	174	8.0	Carbohydrate transport and metabolism
E	134	6.2	Amino acid transport and metabolism
F	68	3.1	Nucleotide transport and metabolism
H	98	4.5	Coenzyme transport and metabolism
I	62	2.9	Lipid transport and metabolism
P	104	4.8	Inorganic ion transport and metabolism
Q	29	1.3	Secondary metabolites biosynthesis, transport and catabolism
R	285	13.2	General function prediction only
S	125	5.8	Function unknown
-	1,825	47.6	Not in COGs
